# An Exploratory Assessment of Self-Reported Satisfaction with Infrastructure and Out-of-Home Activities for People with Vision Impairments

**DOI:** 10.3390/vision7030058

**Published:** 2023-09-02

**Authors:** Mohammad M. Hamed, Maisaa A. Masoud

**Affiliations:** 1 Engineering Faculty, Civil Engineering Department, Isra University, Queen Alia International Airport Road, Amman 11118, Jordan; 2 Vision Rehabilitation Center, German Jordanian University, Amman 11180, Jordan; maysa.mitane@gju.edu.jo

**Keywords:** vision impairment mobility, infrastructure, unobserved heterogeneity, ordered probit model

## Abstract

Purpose: The purpose of this study is to assess the satisfaction levels of people with VI with regard to infrastructure and outdoor activities. Furthermore, this study aims to develop an assessment model for the levels of difficulty in using public transport. Methods: Participants in a standardized survey questionnaire included 74 participants with VI. Three assessment-ordered probit models were estimated based on self-reported responses. Results: Estimation results revealed that the use of public transport is extremely difficult for 83.47% of older participants. In addition, 84.2% of people with albinism have extreme difficulty using public transport. Furthermore, 53.98% of people with restricted horizontal and vertical fields face extreme difficulty using public transport. There was dissatisfaction with outdoor activities among 97.40% of people with macular disease. The results show that 51.70% of people with normal or near-normal horizontal visual fields and restricted vertical planes are satisfied with their level of outdoor activity while 72.65% of people with retinal diseases expressed dissatisfaction with the existing infrastructure. Conclusion: This study revealed that the experiences of people with VI are heterogeneous and depend on their eye condition, access to assistive technology, and socioeconomic characteristics. Results clearly show evidence of heterogeneity among individuals with VI. The combination of horizontal and vertical restrictions yields random parameters, underscoring the heterogeneous experiences of people with VI, influenced by their eye condition and access to assistive devices. Our results have important implications for developing targeted interventions to enhance the mobility of people with VI.

## 1. Introduction

Key factors such as aging, genetic predispositions, lifestyle, infectious diseases, and other health conditions are responsible for visual impairments (VI) [1]. It is estimated that approximately 12 million people (individuals older than 50 years old) have some degree of VI, or blindness, globally [1,2]. Visual impairment continues to be the predominant cause of disability among older adults [3]. Visual impairment can severely inhibit the independence and functioning of people with VI [3,4,5]. Their mobility, access to employment opportunities [6], and social interaction are seriously hampered [7,8]. More specifically, they are faced with temporal and spatial constraints that restrict their choices of destinations and out-of-home activities [9].

Persons with VI face various challenges that also affect their mental health and social well-being. Past research suggests that they are more receptive to stress and depression due to lower life satisfaction [10,11]. Furthermore, the absence of facial recognition can worsen social barriers [12]. Low et al. [13] argue that access to information, inconsistent infrastructure, and limited staff support contribute to the wide-ranging challenges faced by people with VI. Moreover, the lack of access to public transport aggravates the limited mobility of working-age persons with VI [6,13,14]. Restriction in mobility and the inability to travel independently to participate in out-of-home activities are two of the most significant challenges faced by people with VI [15]. They face wide-ranging aspects of mobility, such as navigating unfamiliar locations, using public transport without assistance, and detecting and avoiding hazards. Compared to those without VI, people with VI have slower walking speeds, fall very often, and have trouble navigating their environments [16,17].

People with VI are assessed using measures of contrast sensitivity, visual acuity, and peripheral visual field. Assessments of visual function have been found to be related to mobility performance [18,19]. More specifically, narrower visual fields have been shown to worsen mobility for people with reduced visual fields [20,21,22,23]. Peripheral visual field loss also has a more negative impact on orientation and mobility (O and M) performance than central visual loss and is associated with an increased risk of falls and tripping over obstacles [17,24]. Moreover, the loss of the central visual field prevents individuals from performing activities that entail fine details, such as reading various signs and recognizing depth [25]. To achieve safer mobility, individuals with VI may use assistive devices such as white canes, guide dogs, GPS navigation tools, and cognitive mapping skills.

Participating in outdoor activities is a vital aspect of enhancing the physical, emotional, and social well-being of people with VI. Furthermore, sustaining physical activities is crucial in preventing physical and cognitive decline, diminishing the risk of falls [26], and lowering the likelihood of chronic disease [27]. Nevertheless, there are several factors that can contribute to the limited mobility faced by individuals with VI. These factors encompass fear of falling, anxiety about being injured while crossing the street [28], inadequate access to assistive devices, insufficient environmental adjustments, spatial impairment, and age-related concerns. Past research has demonstrated that individuals with VI often experience difficulty identifying barriers, navigating through crowded transport hubs, crossing streets, and recognizing vehicles while utilizing public transportation [29].

In the context of urban areas, the pursuit of independent mobility poses a formidable challenge for individuals with visual impairments (VIs), who frequently experience anxiety about their safety [30]. In developing nations, this situation is further exacerbated by unstructured infrastructure that presents substantial obstacles to autonomous movement [31]. Clarke et al. [32] reported that the conditions of the street and sidewalk exert a profound influence on the walking behaviors of individuals with severe visual impairments, as documented in face-to-face interviews. Equally, Schwartz et al. [28] indicated that drivers exhibit greater compliance in yielding to pedestrians displaying visual aids, such as white canes, as compared to those without such recognizably indicative symbols of visual impairment. Montarzino et al. [5] analyzed the walking patterns of VI individuals and reported that their degree of mobility is significantly impacted by the built environment, including pedestrian crossings and the placement of bus stops. Meanwhile, numerous investigations have examined the accessibility of public transport, with Montarzino et al. [5] indicating that VI individuals are more prone to accidents when walking or using public transport than their sighted counterparts. In contrast, Duncan et al. [33] demonstrated that improved accessibility to public spaces could have a profound influence on the active mobility of VI people in urban environments. Marston and Golledge [34] identified functional barriers, such as a lack of information, as a primary limiting factor that impinges upon the utilization of public transportation by VI individuals.

Persons with VI rely on their residual senses to develop spatial awareness when navigating through the urban landscape, as shown by Kim and Sohn [35]. Auditory output signals and tactile guidance paths with distinct surface differentiation are examples of informative and effective tools for individuals with VI [36,37]. However, bus travel poses several challenges for individuals with VI, including difficulty identifying the correct bus when multiple buses arrive at the stop [13,38], locating the boarding point for the bus [39], and encountering unhelpful drivers [40].

To the best of the authors’ knowledge, no previous studies have assessed the levels of satisfaction with infrastructure and out-of-home activities among people with VI. Thus, the aim of this study is to evaluate these two key components. As public transport is a critical element of infrastructure and a necessity for many individuals with VI in their out-of-home activities, this paper aims to develop an assessment model for levels of difficulty in using public transport. The outputs of this model, i.e., the public transport usage difficulty levels, will serve as explanatory variables in assessing satisfaction with out-of-home activities and infrastructure models. Furthermore, all three components are linked to functional vision, the built environment, and socioeconomic structure.

By taking into account these three components, additional mobility needs for individuals with VI will be identified. If implemented, this could lead to specific and effective policy measures to improve their mobility. This study is unique in its attempt to reveal the broad factors that shape satisfaction levels for people with VI. This study will distinguish itself from prior research by accounting for the issue of unobserved heterogeneity (UH), which may result from deviations in the weight of unknown factors across the participants. The modeling approach employed in this study will adopt the random parameters (RPs) ordered probit models that explicitly account for the unobserved characteristics varying across observations [41]. Specifically, three random parameter-ordered probit models would be estimated. The empirical setting and dataset creation will be explained in the following section, followed by a description of the study methodology. Next, the estimation results will be presented and discussed, followed by a Section 5 that summarizes this study’s findings and recommendations.

## 2. Materials and Methods

### 2.1. Participants

A survey was undertaken with the participation of 74 individuals with VI who had prior experience visiting the Vision Rehabilitation Center (VRC) at the German Jordanian University (GJU). The center is situated in Amman, the capital of Jordan, and it served as the focal point for this study. Among a larger group of 300 individuals with VI who were invited to participate, these 74 individuals volunteered for the survey. The age range of the participants in this study varied from 18 to 85 years old, with 36.5% falling between 18 and 35 years old and 28.4% falling between 35 and 50 years old. On the other hand, the age group between 50 and 65 years represents the smallest segment, making up 16% of the sample. During participants’ visits to the VRC, visual function assessments were conducted for each participant. The assessments included measurements of binocular visual parameters (visual acuity, peripheral visual field, and contrast sensitivity). Binocular measurements are considered more valuable for assessing and predicting functional performance, as individuals with low vision tend to use both eyes together in their daily activities. Therefore, binocular assessment provides a more comprehensive understanding of functional abilities and quality of life compared to monocular assessment [42,43,44].

The Lea Symbol illuminated Chart was used to measure binocular best corrected visual acuity (VA), starting from a distance of 3 m. According to the World Health Organization World Report on Vision in 2019 [1], distance vision impairment is categorized into four subcategories: mild (0.3 ≤ VA < 0.5), moderate (0.3 > VA ≥ 0.1), severe (0.1 > VA ≥ 0.05), and blindness with VA worse than 0.05. Each subject’s VA was recorded within one of these categories. Contrast sensitivity (CS) was evaluated using the Lea Low Contrast Flip Chart Test at various contrast levels (25%, 10%, 5%, and 2.5%). The results of contrast sensitivity were recorded as either normal or poor. The peripheral binocular visual field (VF) was examined using the confrontational technique with two sticks measuring 22 cm in length. One stick had a black-colored head, while the other had an orange-colored head, with each head having a diameter of 3.5 cm. We recorded restrictions in the four main directions (Right, Left, up, and down). The normal horizontal field is considered to be 180°, while the vertical field is 135°. To determine the binocular peripheral restriction in the two planes, the horizontal restriction was calculated by summing the restrictions in the Right and Left directions, while the vertical restriction was calculated by summing the restrictions in the Up and Down directions. As shown in Table 1, the horizontal and vertical visual field restrictions resulted in four categories. 

### 2.2. Mobility Questionnaire

The research team developed the questionnaire using the Independent Mobility Questionnaire (IMQ [23,24]) as the standard reference. In addition, the team incorporated further questions to align with the research goals. The questionnaire consisted of four main sections. The first section focused on sociodemographic information and consisted of 24 questions. The second section addressed visual functions. The third section addressed the independent mobility questionnaire, which consisted of three parts. In the first part of the survey, subjects were asked to name three factors that contributed most to stress in mobility situations. The second part had 35 questions relating to specific mobility situations. Subjects rated the level of difficulty they experienced in each situation on a scale of 1 to 5. Thus, a rating of one indicates “no difficulty”, while a rating of five indicates “extreme difficulty”. Mobility behavior was the subject of the third part, which consisted of 14 questions. Finally, nine questions were added to measure the subjects’ perceptions of mobility challenges and satisfaction. Data for this study were collected from the subjects via telephone interviews conducted by the low-vision specialist at the VRC. We explained to the subjects the purpose and questionnaire of the study. They were given the opportunity to participate in the survey on their own initiative. All subjects gave their verbal consent and approval to participate in this study. Each telephone interview took 25 to 35 min. This study adhered to the strict principles outlined in the Declaration of Helsinki and received the approval of the research ethics committee at GJU earlier. These measures ensured that the ethical implications of all research activities were carefully considered and that all participants’ dignity and rights were respected.

### 2.3. Random Parameters Ordered Probit Model Formulation

To assess the mobility satisfaction of VI persons, we focused on three questions identified in the survey questionnaire. The questions used in this study were: 1. The level of difficulty associated with the use of the public transport system: subjects were asked to rate their level of difficulty using a five-point Likert scale. The question asked, “Please rate the level of difficulty when using the public transport system using the response options stated below”—“Extremely Satisfied”; “Satisfied”; “Neutral”; “Dissatisfied”; and “Extremely Dissatisfied”. 2. The level of satisfaction with current out-of-home activities: subjects were asked to express their satisfaction level by using a five-point Likert scale. The question asked, “Please rate your level of satisfaction with current out-of-home activities using the response options stated below”—“Extremely Satisfied”; “Satisfied”; “Neutral”; “Dissatisfied”; and “Extremely Dissatisfied”. 3. The level of satisfaction with the prevailing infrastructure and environmental adaptation (streets, sidewalks, pedestrian crossings, signs, etc.): participants were asked to state their satisfaction level using a five-point Likert scale. Response options included “extremely satisfied”, “satisfied”, “neutral”, “dissatisfied”, and “extremely dissatisfied”.

The outputs obtained from the first model (level of difficulty when using public transport) were considered explanatory variables in the second and third models. This approach allowed us to explore the relationship between the respondents’ perceived difficulty in using public transport and their satisfaction with out-of-home activities and infrastructure/environmental adaptation. By employing these dependent variables and analyzing their relationships, we aimed to obtain valuable insights into the mobility satisfaction of individuals with vision impairment. Given the ordinal, discrete nature of the available responses, an ordered probability modeling approach is an appropriate choice [41,45,46,47]. The ordered probability model is specified by expressing an unobserved latent continuous variable, z, for each subject n as the linear function [41,45],

(1) $$z_{n}=\beta X_{n}+\varepsilon_{n}$$

where β represents a vector of estimable parameters, $X_{n}$ represents the vectors with the potential independent variables for subject n, and $\varepsilon_{n}$ is a random error term assumed to be normally distributed in the probit formulation. The collected ordinal responses $y_{n}$ are defined as [41,45],

(2) $$\begin{array}{ll} y_{n} & =1{\;\mathrm{if}\;z}_{n}\leq 0\\ & =2\;\mathrm{if}\;0<z_{n}\leq \mu_{1}\\ & =3{\;\mathrm{if}\;\mu }_{1}<z_{n}\leq \mu_{2}\\ & =4{\;\mathrm{if}\;\mu }_{2}<z_{n}\leq \mu_{3}\\ & =5{\;\mathrm{if}\;z}_{n}>\mu_{3} \end{array}$$

where $\mu_{1}$, $\mu_{2}$, and $\mu_{3}$ represent a vector of estimable threshold parameters for the defined five categories and estimated with the vector of estimable parameters β [41,42,43,44,45]. To account for the effect of unobserved factors varying across all participants, random parameters are introduced. The random parameters modeling approach involves the estimation of subject-specific parameter vectors for the response variables to deal with unobserved heterogeneity [41]. The changes in the effects of observable features can be modeled by introducing specific parameter vectors, $\beta_{k_{n}}$, into the vector of estimable parameters, where [45],

(3) $$\beta_{k_{n}}=\beta_{k}+\omega_{k_{n}}$$

where $\beta_{k}$ is the parameter estimate for explanatory variable k for subject n, and $\omega_{k_{n}}$ is a random term following the standard normal distribution (with a mean equal to zero and a standard deviation $\sigma^{2}$). To compute the effect of any variable in the vector $X_{n}$ on each response category $y_{n}$, the marginal effects are required [41]. To compute the mean marginal effects for the variable in the vector $X_{n}$, all five choice probabilities can be found as [45],

(4) $$\begin{array}{l} P\left(y=1\right)=\Phi \left(-\beta X_{n}\right)\\ P\left(y=2\right)=\Phi \left(\mu_{1}-\beta X_{n}\right)-\Phi \left(-\beta X_{n}\right)\\ P\left(y=3\right)=\Phi \left(\mu_{2}-\beta X_{n}\right)-\Phi \left(\mu_{1}-\beta X_{n}\right)\\ P\left(y=4\right)=\Phi \left(\mu_{3}-\beta X_{n}\right)-\Phi \left(\mu_{2}-\beta X_{n}\right)\\ P\left(y=5\right)=1-\Phi \left(\mu_{3}-\beta X_{n}\right) \end{array}$$

where Φ(.) is the cumulative normal distribution. Finally, the mean marginal effects (computed at the means of the random parameters) can be computed as [45],

(5) $$\begin{array}{l} \frac{\partial P\left(y=1\right)}{\partial X_{n}}=-\left[\phi \left(-\beta X_{n}\right)\right]\beta \\ \frac{\partial P\left(y=2\right)}{\partial X_{n}}=-\left[\phi \left(\mu_{1}-\beta X_{n}\right)-\phi \left(-\beta X_{n}\right)\right]\beta \\ \frac{\partial P\left(y=3\right)}{\partial X_{n}}=-\left[\phi \left(\mu_{2}-\beta X_{n}\right)-\phi \left(\mu_{1}-\beta X_{n}\right)\right]\beta \\ \frac{\partial P\left(y=4\right)}{\partial X_{n}}=-\left[\phi \left(\mu_{3}-\beta X_{n}\right)-\phi \left(\mu_{2}-\beta X_{n}\right)\right]\beta \\ \frac{\partial P\left(y=5\right)}{\partial X_{n}}=\left[\phi \left(\mu_{3}-\beta X_{n}\right)\right]\beta \end{array}$$

where ϕ(.) is the normal density. It should be noted that there exist several advanced methodologies that account for unobserved heterogeneity depending on the structure of the models and the type of data (e.g., random threshold models, HOPIT, zero-inflated ordered models, latent class models, correlated random parameter models, grouped random parameter models, and random parameter models with heterogeneity in the means and/or variances [48,49,50,51,52,53,54,55,56,57]). It is important to acknowledge that including a substantial number of explanatory variables in the models, coupled with a limited sample size, could potentially lead to overfitting problems. As a result, this could present a caveat to the analysis. However, it is worth noting that this concern is likely to be mitigated to some extent when working with a larger sample size.

## 3. Results

### 3.1. Descriptive Statistics

**Figure 1:**
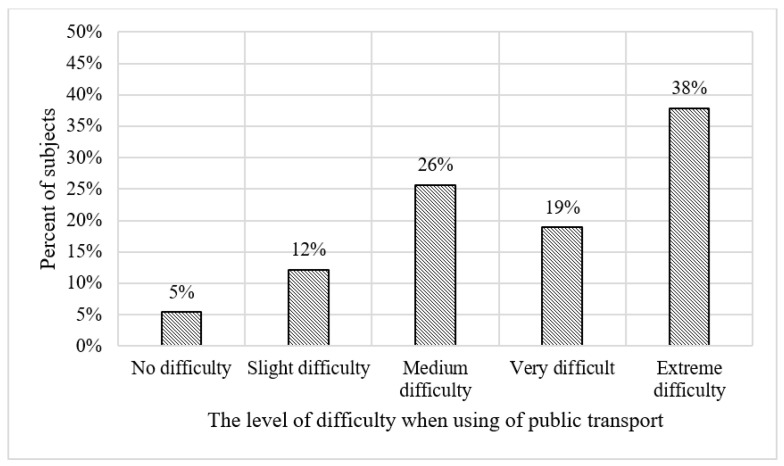
Frequency distribution for the level of difficulty of public transport usage.

**Figure 2:**
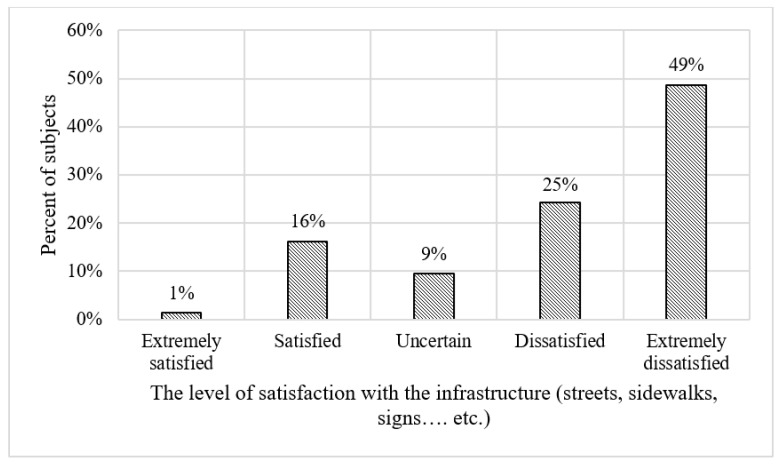
Frequency distribution for the level of satisfaction with the infrastructure.

**Figure 3:**
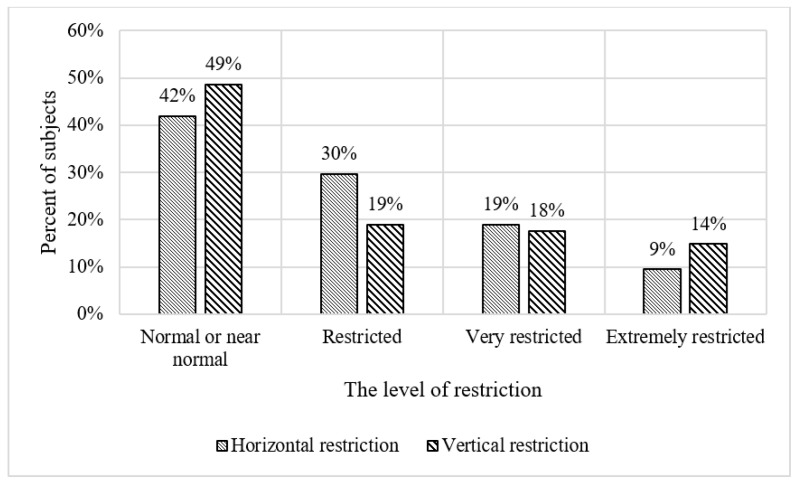
Frequency distribution for the level of restriction.

**Figure 4:**
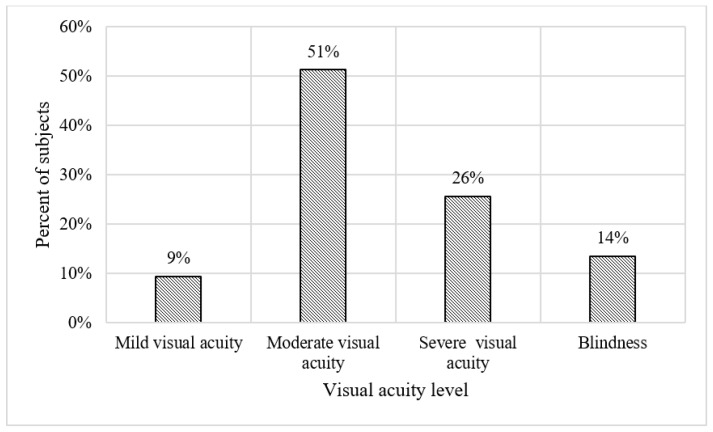
Frequency distribution for visual acuity at different levels.

Table 2 depicts the descriptive statistics of the sample. The survey shows that among the subjects, 53% are male and 47% are female. Figure 1 shows the dispersion of the level of difficulty encountered by subjects when using public transport. The survey shows that 38% reported finding the use of public transportation extremely difficult. Figure 2 shows the spreading of the level of satisfaction with the current infrastructure, including streets, sidewalks, pedestrian crossings, signs, and other related infrastructural elements. Almost half of the subjects expressed extreme dissatisfaction with the existing infrastructure, while only 16% reported being satisfied. This dissatisfaction is likely to stem from the infrastructure’s inability to effectively meet the needs of people with VI. Figure 3 and Figure 4 show the vision functional assessment results obtained from our sample of subjects. 

Figure 3 shows the horizontal and vertical peripheral vision of the subjects. It can be seen that 42% of the subjects had normal or near-normal horizontal peripheral vision, while 9% experienced extreme restrictions in this aspect. Similarly, in terms of vertical peripheral vision, 49% of the subjects showed normal or near-normal vision, whereas 14% experienced severe restrictions. Figure 4 shows the distribution of visual acuity at various levels. The results indicate that 51% of the subjects had moderate visual acuity, while 9% had mild visual acuity. Finally, the data show that 80% of the subjects exhibited poor contrast sensitivity.

### 3.2. Random Parameters Ordered Probit Modes Results

Table 3, Table 4 and Table 5 present the estimates and marginal effects of the Random Parameters Ordered Probit models for the level of difficulty when using public transport, levels of satisfaction with out-of-home activities, and levels of satisfaction with the prevailing infrastructure (streets, sidewalks, pedestrian crossings, signs, etc.), respectively. Our estimation results show several statistically significant parameters with plausible signs. All parameter estimates have statistical significance at the 90%, 95%, or 99% confidence levels. The results show significant unobserved heterogeneity, as each model has variables that generate random parameters with statistically significant standard deviations. More specifically, the first model (Table 3) shows four significant random parameters (statistically significant standard deviations), clearly indicating that the effect of these variables varied significantly across individuals with VI. The second model (Table 4) shows four significant random parameters (statistically significant standard deviations). The third model (Table 5) shows three significant random parameters (statistically significant standard deviations). Furthermore, all three models demonstrate reasonably good overall statistical fit, as shown by McFadden’s $\rho^{2}$ values of 0.378 (Table 3), 0.346 (Table 4), and 0.455 (Table 5), respectively.

## 4. Discussion

### 4.1. Level of Difficulty When Using Public Transport

Table 3 shows insights into the factors influencing the level of difficulty when using public transport, including demographic and socioeconomic characteristics, visual function characteristics, and mobility characteristics. The results indicate the significance of demographic and socioeconomic factors in determining this level of difficulty. One significant finding is related to age, where older individuals show varying levels of difficulty when using public transport. The age variable (1 if greater than 50 years old; 0 otherwise) generated a random parameter with a mean value of 7.844 and a standard deviation of 8.064 (Table 3). This result indicates that 83.47% of older individuals experience extreme difficulty using public transport, while 16.53% experience lower levels of difficulty. Crudden et al. [58] reported that the challenges faced by older individuals in using public transport might be attributed to feelings of vulnerability and a greater reliance on assistance from others thus leading to increased stress and anxiety. Fiedler [59] reported that age-related factors such as vision and hearing loss, functional limitations due to various diseases, physical exertion, cognitive limitations, and psychological factors contribute to mobility challenges. Crews et al. [60] indicated that people over 65 with VI experience significantly greater difficulties in performing basic physical and social activities compared to their peers without vision impairment. Socioeconomic explanatory variables also turned out to play a part in the level of difficulty when using public transport. Variables such as the total number of vehicles owned by the household, unemployment status, and total household income were found to have a significant impact on this level of difficulty.

The estimation results show that retinal diseases, such as macular disease and albinism, significantly influence the level of difficulty faced by people with VI when using the public transport system. These people often struggle with reading street signs and identifying landmarks due to their retinal diseases. Montarzino et al. [5] indicated that people with age-related macular degeneration face challenges due to unclear service information, timetables, and poor visibility of destinations. Hazel et al. [4] indicated that individuals with age-related macular degeneration have poorer reading skills due to decreased contrast sensitivity. Similarly, people with albinism experience multiple problems and limitations when using public transport, including photophobia, discomfort from glare, reduced distance vision, and a lack of depth perception [61]. Furthermore, the ophthalmological diagnosis indicator variable (1 if albinism; 0 otherwise) generated a random parameter with a mean value of 10.712 and a standard deviation of 10.685 (Table 3). This implies that 84.20% of people with albinism find it extremely difficult to use public transport, while 15.80% experience lower levels of difficulty.

The severity and manifestations of ocular characteristics in albinism can vary among individuals. Variations include reduced visual acuity and contrast sensitivity, high refractive errors, nystagmus, amblyopia, and photophobia [62,63]. Our findings align with similar results reported by [64]. Our results also show that various visual function characteristics significantly affect the level of difficulty people face when using public transport. Visual acuity, contrast sensitivity, and visual field have been established as important factors that correlate with mobility performance [18,19,65]. Severe visual acuity loss, as indicated in Table 3, is found to have a positive and significant parameter. This suggests that individuals with severe visual acuity loss are more likely to experience extreme difficulty when using public transportation. With low visual acuity, perceiving the size, shape, and details of objects becomes challenging. Distinguishing colors accurately and making distance judgments become very difficult tasks. Furthermore, navigating crowded environments such as bus terminals can pose significant challenges. Clinical studies have also indicated a strong connection between visual acuity and mobility [66,67,68,69]. The results reported in our study align with past research, highlighting the impact of visual acuity on individuals’ ability to use public transport effectively.

The estimation results also show that people with poor contrast sensitivity, as shown in Table 3, are highly likely to experience extreme difficulties when using public transport modes. Contrast sensitivity plays a crucial role in object detection and recognition, making mobility more challenging. Black et al. [64] indicated that the difficulties in recognizing obstacles and the slower walking speed are common consequences of poor contrast sensitivity. Our results are consistent with earlier research findings [29,70,71,72]. Our results (Table 3) also show that the estimated parameter for extremely restricted horizontal and restricted vertical peripheral visual field (PVF) is positive and significant. This indicates that people with narrower visual fields experience more difficulties in mobility performance, as supported by previous studies [21,22,23,73]. Marron and Bailey [70] reported that PVF loss has a greater impact on orientation and mobility compared to central visual field loss. PVF is also associated with an increased risk of falls and tripping over obstacles [17,24]. Past research indicated that restricted PVF not only affects postural stability but also impairs motion assessment and the ability to avoid peripheral obstacles [74,75]. In the case of an extremely restricted horizontal plan, the right and left fields of vision are limited, which can hamper the perception of information relevant to public transport. Similarly, an extremely restricted vertical plan limits the upper and lower fields of vision, affecting the ability to detect lower and upper obstacles and elevated signs [24]. Our findings are in line with the work of Wan et al. [76].

Moreover, the results demonstrate that restricted horizontal and vertical plans of the peripheral visual field generated a random parameter with a mean value of 1.616 and high standard deviations of 16.180. This suggests that 53.98% of people with this restriction face extreme difficulty when using public transport, while 46.02% experience lower levels of difficulty. This finding clearly highlights the heterogeneity within the visually impaired population thus indicating that the effects of specific factors can vary among individuals. Loetscher et al. [77] reported that compensatory scanning behaviors employed by people with visual field restrictions might partially compensate for restricted vision and improve task performance.

The estimation results in Table 3 indicate that the absence of orientation and mobility training increases the probability of experiencing extreme difficulty when using public transport modes. Orientation and mobility training are instrumental in enhancing the ability of persons with VI to navigate their surroundings safely, interpret sound cues, acquire skills to identify objects, and navigate unfamiliar environments [78,79]. Our outcomes are in line with the research by Crudden et al. [58], which demonstrates that orientation and mobility training reduce mobility-related stress for individuals with vision impairments. Our estimation results also show that the absence of environmental adaptations for people with VI increases the likelihood of facing significant difficulties when using public transport.

### 4.2. Level of Satisfaction with Out-of-Home Activities

In Table 4, we present the levels of satisfaction with out-of-home activities of people with VI. Estimation results clearly indicate that these satisfaction levels are shaped by demographic and socioeconomic characteristics, visual functional characteristics, and mobility characteristics. The parameter estimates for all ophthalmological diagnoses (Table 4) are positive and significant. This clearly suggests that people diagnosed with retinitis pigmentosa and corneal diseases are more likely to express dissatisfaction or extreme dissatisfaction with their current out-of-home activities. Our outcomes are in line with previous research by Black et al. [64] and Spadea et al. [80].

The estimation results also show that people with macular diseases express dissatisfaction with their current out-of-home activities. This clearly suggests that people with macular diseases are less likely to participate in outdoor activities due to concerns regarding their safety in unfamiliar environments. However, this generated a random parameter with a mean value of 14.422 and a standard deviation of 7.423. This indicates that 97.40% of individuals with macular diseases were dissatisfied with the prevailing level of out-of-home activities, while only 2.60% expressed satisfaction. Our result is in line with the work by Hazel et al. [4], who reported that challenges faced by people with macular diseases are likely related to their limited reading skills, which result from poor contrast sensitivity.

Results presented in Table 4 also show that individuals who use assistive devices such as low-vision filters are more likely to express satisfaction with their out-of-home activity levels. Wearing low-vision filters has been shown to improve mobility by mitigating the negative effects of sunlight and high illumination, reducing glare sensitivity, alleviating photophobia, and minimizing eye discomfort [81]. Moreover, previous research studies [81,82,83] have reported significant improvements in reading ability following the use of low-vision filters, which can enhance individuals’ participation in out-of-home activities. The results in Table 4 also show that people with poor contrast sensitivity are more likely to express dissatisfaction with their out-of-home activity levels. It has been reported that poor contrast sensitivity can lead to difficulties in recognizing objects and distinguishing shades, which may result in individuals walking more slowly [64]. The slower walking pace can impede the person’s ability to navigate efficiently and engage in activities outside their homes. Our findings are in line with previous research studies that have identified contrast sensitivity as a significant predictor of mobility performance [69,70,84,85,86].

Our results also show that peripheral visual fields with a variety of combinations of horizontal and vertical restriction plans resulted in the production of random parameters, as shown in Table 4. The observed randomness may be attributed to the manner in which subjects scan their surrounding environment to identify people and objects [87]. Table 4 also shows that people with normal or near-normal horizontal plans and restricted vertical plans show a higher likelihood (51.70%) of being satisfied with their out-of-home activity levels. In contrast, 48.3% of these people express dissatisfaction with their activity levels. facing significant challenges when navigating crowded environments, traversing stairs, or locating public restrooms expressed their dissatisfaction with their level of outdoor activities. Interestingly, even individuals who successfully utilize Taxi-App services through their smartphones express dissatisfaction with their out-of-home activities. This may be attributed to the high cost associated with Taxi-App services. Additionally, people who encounter extreme difficulty when using public transport (as identified in the first model’s output) express dissatisfaction with their current out-of-home activity levels. Our findings line up with previous research conducted by Low et al. [13], highlighting the limited accessibility of the public transport system for people with visual impairments.

Our estimation results also show that people who are content with their mobility performance may not perceive the need for orientation and mobility training. This finding lines up with previous studies by Bibby et al. [18] and Beggs [88], which concluded that a combination of visual functional outcomes and mobility difficulties could serve as a determining factor for the necessity of O&M training. This clearly implies that those who are satisfied with their mobility performance are less likely to seek out O&M training, while individuals who are unaware of mobility training exhibit lower levels of satisfaction. Finally, results show that people who participate in only one out-of-home activity per week are more likely to experience dissatisfaction or extreme dissatisfaction with their out-of-home activities. Limiting oneself to a single out-of-home activity per week may signify underlying issues related to mobility and social engagement. Similar results were reported by [89] in their study on people with Age-related macular degeneration (AMD).

### 4.3. Level of Satisfaction with the Infrastructure

Results presented in Table 5 show that people diagnosed with retinitis pigmentosa are significantly more likely to experience extreme dissatisfaction with the existing infrastructure. In fact, this disease contributes to a 0.546 increase in the probability of dissatisfaction (Table 5). Persons with retinitis pigmentosa often encounter difficulties in making accurate distance judgments, navigating sidewalks, and recognizing signs, especially when exposed to sunlight and substantial contrast variations between different environments. Ref. [54] Similarly, other retinal diseases such as diabetic retinopathy, retinopathy of prematurity, and retinal detachment also increased the likelihood of extreme dissatisfaction with existing infrastructure. In fact, these retinal diseases produced a random parameter with a mean of 3.616 and a standard deviation of 6.004. This indicates that 72.65% of individuals with these conditions expressed dissatisfaction, while 27.35% reported satisfaction. The observed randomness can be attributed to significant differences in infrastructure across various locations in Jordan.

Estimation results also indicate that people with albinism tend to be satisfied with the current infrastructure. This variable generated a random parameter with a mean of −2.541 and a standard deviation of 2.583. This clearly indicates that 16.26% of people expressed dissatisfaction, while the majority (83.74%) reported satisfaction. This outcome can be attributed to the non-progressive nature of albinism and the absence of vision deterioration over time. Additionally, individuals with albinism typically have less restricted visual fields, allowing them to become familiar with their surroundings and potentially experience greater satisfaction. Similarly, wearing low-vision filters, as shown in Table 5, generated a random parameter with a mean of 1.650 and a standard deviation of 2.082. The majority of people wearing low-vision filters (78.60%) expressed their dissatisfaction with the infrastructure, while 21.40% reported satisfaction. Although assistive devices such as low-vision filters are helpful for mobility, they alone are insufficient to ensure adequate mobility. Accessible infrastructure remains crucial for individuals with visual impairments. A recent study conducted in Jordan by Al-Khudair et al. [90] assessed the accessibility of public infrastructure for individuals with visual and mobility disabilities. The study revealed significant challenges, particularly concerning streets, sidewalks, and government and private sector buildings, leading participants to express dissatisfaction with the accessibility of public infrastructure.

The estimation results in Table 5 show important insights regarding the association between VI and the level of satisfaction with the infrastructure: Firstly, people with severe visual acuity show a significant increase (0.670) in the likelihood of being extremely dissatisfied with the infrastructure. Low visual acuity directly affects distance judgment and mobility [91]. Secondly, individuals with restricted horizontal and severely restricted vertical visual fields express extreme dissatisfaction with the infrastructure. The interaction with infrastructure elements [92,93,94], such as streets, sidewalks, and pedestrian crossings, is closely tied to the field of vision. We note here that horizontal restrictions hinder the recognition of cars, objects, and pedestrians on the streets, while vertical restrictions affect walking speed, descending steps, and obstacle recognition [24]. Lastly, the absence of environmental adaptations for people with visual impairments significantly increases the likelihood of extreme dissatisfaction with the infrastructure. Kim and Sohn [35] reported a similar outcome.

## 5. Conclusions

The objective of this study was to assess the satisfaction levels of individuals with visual impairments regarding their out-of-home activities and the accessibility of existing infrastructure. By considering the heterogeneity of visual impairments, the analysis examined the impact of vision functional assessment, orientation and mobility training, and the built environment. Our results show that ophthalmological diagnoses such as albinism and macular diseases produce random parameters with a statistically significant standard deviation. For example, individuals with albinism had varying levels of satisfaction with the available infrastructure and encountered difficulties while using public transportation. Similarly, people with macular disease showed differing levels of satisfaction with outdoor activities. The findings highlight the heterogeneity of individuals with VI and their unique needs. Furthermore, individuals with ophthalmological diagnoses such as retinitis pigmentosa, macular diseases, and corneal diseases expressed dissatisfaction with their activities outside their homes and the infrastructure. The results also indicate that access to and usage of assistive devices such as special low-vision filters are crucial for using public transport.

Our results indicate that individuals with severe visual acuity experience significant difficulty using public transport and express dissatisfaction with infrastructure. Similarly, individuals with restricted peripheral vision fields express dissatisfaction with out-of-home activities and infrastructure. Furthermore, the combination of horizontal and vertical restrictions yields random parameters with a highly significant standard deviation, underscoring the heterogeneous experiences of people with VI, influenced by their eye condition and access to assistive devices. Our results have important implications for developing targeted interventions to enhance mobility for people with VI, especially in light of the diverse and complex challenges they face in their daily activities.

Our findings show that public transport usage is facilitated for those who are proficient in navigating unfamiliar environments, while individuals facing challenges in crowded situations encounter greater difficulty. Furthermore, people who experience difficulty in crowded environments, using public transport, maneuvering steps, and locating public restrooms exhibit lower satisfaction levels with their out-of-home activities. Those experiencing difficulty descending stairs or walking in low-light conditions also report lower satisfaction levels with infrastructure. Of particular significance is the scarcity of environmental adaptations for individuals with low vision, further compounding the challenges of public transport usage and depressing satisfaction levels with infrastructure. These findings emphasize the urgent need to augment environmental adaptations and enhance infrastructure accessibility for individuals with visual impairments, ultimately promoting greater mobility and quality of life.

The estimation results reported in this paper have important implications. Firstly, assistive devices and low-vision filters must be made more accessible and available to those in need. Secondly, infrastructure accessibility for individuals with visual impairment (VI) needs to be improved, as 73.20% of participants were dissatisfied with the current infrastructure. Thirdly, our findings can aid vision rehabilitation specialists in assessing the need for O&M training for people with VI. Lastly, there is a pressing need for comprehensive visual assessments for individuals with VI, as peripheral visual field loss was significantly correlated with poor contrast sensitivity. This paper is limited in several ways. Firstly, the cross-sectional nature of this study precludes investigation into how factors affecting mobility issues evolve over time, which is essential for understanding the correlation between heterogeneous causes of visual loss and mobility issues. Secondly, the sample used is not fully representative of individuals with visual impairments (VIs) as a whole. Thirdly, the generalizability of this study’s findings to persons with VIs in developed countries may be constrained by varying physical environments, differential accessibility to resources such as assistive devices, and divergent infrastructure and public transport systems.

## Figures and Tables

**Table 1:** Horizontal and vertical plane restriction levels.

Category	Horizontal Restriction	Vertical Restriction
Normal or near to normal	0°–20°	0°–20°
Restricted	>20°–90°	>20°–70°
Very restricted	>90°–160°	>70°–115°
Extremely restricted	>160°–180°	>115°–135°

**Table 2:** Description and descriptive statistics of variables available for modeling.

Variable Description	Mean	Standard Deviation	Variable Description	Mean	Standard Deviation
Demographic and socioeconomic Characteristics			Moving about in crowded situations (1 if extreme difficulty; 0 otherwise)	0.392	0.492
Age of subject (1 if between than 18 and 35 years old; 0 otherwise)	0.365	0.481	Walking at night (1 if extreme difficulty; 0 otherwise)	0.419	0.497
Age of subject (1 if between than 35 and 50 years old; 0 otherwise)	0.284	0.451	Using public transport (1 if extreme difficulty; 0 otherwise)	0.378	0.488
Age of subject (1 if between than 50 and 65 years old; 0 otherwise)	0.162	0.369	Walking down steps (1 if extreme difficulty; 0 otherwise)	0.135	0.344
Age of subject (1 if greater than 65 years old; 0 otherwise)	0.189	0.392	Finding restrooms in public places (1 if extreme difficulty; 0 otherwise)	0.365	0.485
Subject gender (1 if male; 0 otherwise)	0.527	0.503	Usage of taxi-App through smart phones (1 if no difficulty; 0 otherwise)	0.108	0.313
Number of vehicles in household	1.081	0.772	Fallen incidence (1 if fallen last year; 0 otherwise)	0.784	0.414
Total monthly household income (1 if from USD 570 to 1127 USD; 0 otherwise)	0.514	0.503	Fallen incidence (1 if did not fall last year; 0 otherwise)	0.216	0.414
Total monthly household income (1 if from USD 2254 to 2817 USD; 0 otherwise)	0.041	0.199	Mobility limitation due to vision impairment (1 if no; 0 otherwise)	0.270	0.447
Subject educational level (1 if a university degree holder; 0 otherwise)	0.378	0.488	Mobility training (1 if did not undertake any mobility training; 0 otherwise)	0.919	0.275
Subject occupation (1 if retired; 0 otherwise)	0.176	0.383	Reason for not undertaking mobility training (1 if unaware of mobility training; 0 otherwise)	0.392	0.492
Subject occupation (1 if unemployed; 0 otherwise)	0.257	0.440	Reason for not undertaking mobility training (1 if no environmental adaptation for persons with low vision outside the home; 0 otherwise)	0.027	0.163
Ophthalmological diagnoses (1 if retinitis pigmentosa; 0 otherwise)	0.267	0.447	Subject belief that the ability to travel on foot alone is less than those with normal vision (1 if strongly disagree; 0 otherwise)	0.068	0.253
Ophthalmological diagnoses (1 if macular diseases; 0 otherwise)	0.267	0.447	Pursuing out-of-home activities (1 if pursuing two to three out-of-home activities per week; 0 otherwise)	0.243	0.432
Ophthalmological diagnoses (1 if albinism; 0 otherwise)	0.108	0.313	Pursuing out-of-home activities (1 if pursuing less than one out-of-home activity per week; 0 otherwise)	0.122	0.329
Ophthalmological diagnoses (1 if other retinal diseases; 0 otherwise)	0.160	0.371	Satisfaction level with public transport infrastructure in your area (1 if satisfied; 0 otherwise)	0.122	0.329
Ophthalmological diagnoses (1 if corneal diseases; 0 otherwise)	0.066	0.253	Challenges when using public transport (1 if non-existence of environmental adaptations for people with low vision; 0 otherwise)	0.622	0.488
Ophthalmological diagnoses (1 if optic atrophy diseases; 0 otherwise)	0.051	0.228			
Ophthalmological diagnoses (1 if glaucoma diseases; 0 otherwise)	0.081	0.275	Level of difficulty when using of public transport (1 if no difficulty; 0 otherwise)	0.054	0.228
Onset age of low vision (1 if greater than 40 years; 0 otherwise)	0.216	0.414	Level of difficulty when using of public transport (1 if slight difficulty; 0 otherwise)	0.121	0.329
Usage of assistive devices for O and M (1 if sunglasses; 0 otherwise)	0.324	0.471	Level of difficulty when using of public transport (1 if medium difficulty; 0 otherwise)	0.258	0.440
Usage of assistive devices for O and M (1 if filter; 0 otherwise)	0.203	0.405	Level of difficulty when using of public transport (1 if very difficult; 0 otherwise)	0.189	0.394
Visual function characteristics			Level of difficulty when using of public transport (1 if extreme difficulty; 0 otherwise)	0.378	0.488
Visual acuity (1 if VA < 0.05; 0 otherwise)	0.135	0.344	Level of the satisfaction with prevailing out-of-home activities (1 if extremely satisfied; 0 otherwise)	0.122	0.329
Contrast sensitivity (1 if poor; 0 otherwise)	0.784	0.414	Level of the satisfaction with prevailing out-of-home activities (1 if satisfied; 0 otherwise)	0.446	0.500
Peripheral visual field (1 if horizontal plan ^1^ is normal or near to normal and the vertical plan ^2^ is restricted; 0 otherwise)	0.149	0.358	Level of the satisfaction with prevailing out-of-home activities (1 if uncertain; 0 otherwise)	0.149	0.358
Peripheral visual field (1 if horizontal plan ^1^ is restricted and the vertical plane is restricted; 0 otherwise)	0.135	0.344	Level of the satisfaction with prevailing out-of-home activities (1 if dissatisfied; 0 otherwise)	0.176	0.383
Peripheral visual field (1 if horizontal plan ^1^ is restricted and the vertical plan ^2^ is very restricted; 0 otherwise)	0.054	0.228	Level of the satisfaction with prevailing out-of-home activities (1 if extremely dissatisfied; 0 otherwise)	0.107	0.313
Peripheral visual field (1 if horizontal plan ^1^ is very restricted and the vertical plan ^2^ is extremely restricted; 0 otherwise)	0.068	0.253	Level of satisfaction with the infrastructure (1 if extremely satisfied; 0 otherwise)	0.014	0.116
Peripheral visual field (1 if horizontal plan ^1^ is extremely restricted and the vertical plan ^2^ is extremely restricted; 0 otherwise)	0.068	0.253	Level of satisfaction with the infrastructure (streets, sidewalks, signs…. etc.) (1 if satisfied; 0 otherwise)	0.162	0.371
Mobility characteristics			Level of satisfaction with the infrastructure (streets, sidewalks, signs…. etc.) (1 if uncertain; 0 otherwise)	0.092	0.295
Walking in unfamiliar areas (1 if no difficulty; 0 otherwise)	0.014	0.116	Level of satisfaction with the infrastructure (streets, sidewalks, signs…. etc.) (1 if dissatisfied; 0 otherwise)	0.246	0.432
Moving about in stores (1 if no difficulty; 0 otherwise)	0.162	0.371	Level of satisfaction with the infrastructure (streets, sidewalks, signs…. etc.) (1 if extremely dissatisfied; 0 otherwise)	0.486	0.503
^1^ Horizontal restriction (1) Normal or near normal; 0–20 (2) Restricted; >20–90 (3) Very restricted; >90–160 (4) Extremely restricted; >160–180			^2^ Vertical restriction (1) Normal or near normal; 0–20 (2) Restricted; >20–70 (3) Very restricted; >70–115 (4) Extremely restricted; >115–135		

**Table 3:** Random parameter-ordered probit model of the level of difficulty when using public transport.

Variable Description	Parameter Estimate (t-stat)	Marginal Effects				
		No Difficulty	Slight Difficulty	Medium Difficulty	Very Difficult	Extreme Difficulty
Demographic and socioeconomic characteristics						
Age of subject (1 if greater than 50 years old; 0 otherwise)	7.844 (3.08)	0.000	0.000	−0.626	−0.351	0.977
Standard deviation of the random parameter	8.064 (3.28)					
Onset age of low vision (1 if greater than 40 years; 0 otherwise)	−5.244 (−2.59)	0.000	0.962	−0.943	−0.020	0.000
Number of vehicles in household	1.469 (2.48)	0.000	0.000	−0.039	0.035	0.004
Total monthly household income (1 if from USD 2254 to 2817 USD; 0 otherwise)	9.059 (2.44)	0.000	0.000	−0.025	−0.975	1.000
Subject occupation (1 if unemployed; 0 otherwise)	−2.311 (−1.67)	0.000	0.000	0.269	−0.264	−0.005
Ophthalmological diagnoses (1 if macular diseases; 0 otherwise)	10.341 (3.11)	0.000	0.000	−0.679	−0.321	1.000
Ophthalmological diagnoses (1 if albinism; 0 otherwise)	10.712 (3.10)	0.000	0.000	−0.121	−0.879	1.000
Standard deviation of the random parameter	10.685 (3.23)					
Usage of assistive devices for O&M (1 if sunglasses; 0 otherwise)	−1.538 (−1.71)	0.000	0.000	0.096	−0.093	−0.003
Visual function characteristics						
Visual acuity (1 if 0.05 ≤ VA < 0.1; 0 otherwise)	2.394 (2.24)	0.000	0.000	−0.043	−0.035	0.078
Contrast sensitivity (1 if poor; 0 otherwise)	7.062 (2.99)	0.000	0.000	−0.999	0.952	0.048
Peripheral visual field (1 if horizontal plan ^1^ is restricted and the vertical plan ^2^ is restricted; 0 otherwise)	1.616 (1.04)	0.000	0.000	−0.017	−0.018	0.036
Standard deviation of the random parameter	16.180 (3.27)					
Peripheral visual field (1 if horizontal plan ^1^ is extremely restricted and the vertical plan ^2^ is extremely restricted; 0 otherwise)	6.690 (2.53)	0.000	0.000	−0.030	−0.968	0.999
Mobility characteristics						
Walking in unfamiliar areas (1 if no difficulty; 0 otherwise)	−31.657 (−3.32)	1.000	0.000	−0.003	−0.994	−0.003
Moving about in crowded situations (1 if extreme difficulty; 0 otherwise)	8.357 (3.04)	0.000	0.000	−0.828	−0.142	0.970
Fallen incidence (1 if did not fall last year; 0 otherwise)	−4.343 (−2.72)	0.000	0.000	0.858	−0.846	−0.012
Mobility training (1 if did not undertake any mobility training; 0 otherwise)	5.345 (2.40)	0.000	0.000	−0.992	0.989	0.003
Mobility limitation due to vision impairment (1 if no; 0 otherwise)	−7.246 (−3.11)	0.000	0.000	0.998	−0.891	−0.108
Standard deviation of the random parameter	8.346 (3.27)					
Subject belief that ability to travel on foot alone is less than those with normal vision (1 if strongly disagree; 0 otherwise)	−17.734 (−3.12)	0.368	0.632	0.000	−0.977	−0.023
Satisfaction level with public transport infrastructure in your area (1 if satisfied; 0 otherwise)	−6.799 (−2.69)	0.000	0.000	0.999	−0.990	−0.009
Challenges when using public transport (1 if non-existence of environmental adaptations for persons with low vision; 0 otherwise)	1.424 (1.77)	0.000	0.000	−0.072	0.068	0.004
$\mathrm{Threshold}\;\mu_{1}$	6.583 (2.86)					
$\mathrm{Threshold}\;\mu_{2}$	14.545 (3.26)					
$\mathrm{Threshold}\;\mu_{3}$	20.070 (3.32)					
Model statistics						
Log-likelihood at zero	−106.988					
Log-likelihood at convergence	−66.586					
McFadden’s $\rho^{2}$	0.378					
Number of variables	27					
Number of observations	74					
^1^ Horizontal restriction (1) Normal or near normal; 0–20 (2) Restricted; >20–90 (3) Very restricted; >90–160 (4) Extremely restricted; >160–180	^2^ Vertical restriction (1) Normal or near normal; 0–20 (2) Restricted; >20–70 (3) Very restricted; >70–115 (4) Extremely restricted; >115–135					

**Table 4:** Random parameter-ordered probit model of the level of satisfaction with current out-of-home activities.

Variable Description	Parameter Estimate (t-Stat)	Marginal Effects				
		Extremely Satisfied	Satisfied	Uncertain	Dissatisfied	Extremely Dissatisfied
Demographic and socioeconomic characteristics						
Subject educational level (1 if a university degree holder; 0 otherwise)	1.764 (2.12)	−0.227	0.227	0.000	0.000	0.000
Ophthalmological diagnoses (1 if retinitis pigmentosa; 0 otherwise)	12.963 (3.47)	0.000	−1.000	0.000	0.923	0.077
Ophthalmological diagnoses (1 if macular diseases; 0 otherwise)	14.422 (3.42)	0.000	−1.000	0.000	0.642	0.358
Standard deviation of the random parameter	7.423 (3.59)					
Ophthalmological diagnoses (1 if corneal diseases; 0 otherwise)	16.201 (3.09)	0.000	−0.998	−0.002	0.000	1.000
Usage of assistive devices for O and M (1 if filter; 0 otherwise)	−2.033 (−1.97)	0.000	0.079	−0.079	0.000	0.000
Visual function characteristics						
Visual acuity (1 if VA < 0.05; 0 otherwise)	4.826 (2.81)	0.000	−0.984	0.904	0.080	0.000
Contrast sensitivity (1 if poor; 0 otherwise)	3.198 (3.05)	0.000	−0.128	0.128	0.000	0.000
Peripheral visual field (1 if horizontal plan ^1^ is normal or near to normal and the vertical plan ^2^ is restricted; 0 otherwise)	0.354 (0.28)	0.000	−0.034	0.034	0.000	0.000
Standard deviation of the random parameter	8.327 (3.61)					
Peripheral visual field (1 if horizontal plan ^1^ is restricted and the vertical plan ^2^ is restricted; 0 otherwise)	5.696 (2.86)	0.000	−0.994	0.738	0.256	0.000
Standard deviation of the random parameter	9.892 (3.56)					
Peripheral visual field (1 if horizontal plan ^1^ is very restricted and the vertical plan ^2^ is extremely restricted; 0 otherwise)	−1.583 (−0.87)	0.000	0.042	−0.042	0.000	0.000
Standard deviation of the random parameter	8.221 (3.13)					
Mobility characteristics						
Moving about in crowded situations (1 if extreme difficulty; 0 otherwise)	1.830 (2.89)	0.000	−0.233	0.233	0.000	0.000
Using public transport (1 if extreme difficulty; 0 otherwise)	2.487 (2.32)	0.000	−0.387	0.387	0.000	0.000
Walking down steps (1 if extreme difficulty; 0 otherwise)	5.152 (2.72)	0.000	−0.990	0.860	0.130	0.000
Finding restrooms in public places (1 if extreme difficulty; 0 otherwise)	2.787 (2.59)	0.000	−0.210	0.210	0.000	0.000
Usage of taxi-App through smart phones (1 if no difficulty; 0 otherwise)	4.760 (2.37)	0.000	−0.983	0.892	0.091	0.000
Fallen incidence (1 if did not fall last year; 0 otherwise)	2.165 (1.94)	0.000	−0.438	0.438	0.000	0.000
Mobility training (1 if did not undertake any mobility training; 0 otherwise)	−6.652 (−3.17)	0.000	0.991	−0.289	−0.702	0.000
Reason for not undertaking mobility training (1 if unaware of mobility training; 0 otherwise)	3.121 (2.65)	0.000	−0.528	0.528	0.000	0.000
Pursuing out-of-home activities (1 if pursuing one out-of-home activity per week; 0 otherwise)	2.106 (1.64)	0.000	−0.491	0.491	0.000	0.000
$\mathrm{Threshold}\;\mu_{1}$	12.138 (3.48)					
$\mathrm{Threshold}\;\mu_{2}$	15.894 (3.61)					
$\mathrm{Threshold}\;\mu_{3}$	21.200 (3.65)					
Model statistics						
Log-likelihood at zero	−106.984					
Log-likelihood at convergence	−69.989					
McFadden’s $\rho^{2}$	0.346					
Number of observations	74					
^1^ Horizontal restriction (1) Normal or near normal; 0–20 (2) Restricted; >20–90 (3) Very restricted; >90–160 (4) Extremely restricted; >160–180		^2^ Vertical restriction (1) Normal or near normal; 0–20 (2) Restricted; >20–70 (3) Very restricted; >70–115 (4) Extremely restricted; >115–135				

**Table 5:** Random parameter-ordered probit model of the level of satisfaction with the prevailing infrastructure.

Variable Description	Parameter Estimate (t-Stat)	Marginal Effects				
		Extremely Satisfied	Satisfied	Uncertain	Dissatisfied	Extremely Dissatisfied
Demographic and socioeconomic characteristics						
Subject gender (1 if male; 0 otherwise)	−1.106 (−1.94)	0.000	0.004	0.415	−0.420	0.000
Number of vehicles in household	1.488 (3.11)	0.000	0.000	−0.004	−0.589	0.593
Total monthly household income (1 if from USD 570 to 1127 USD; 0 otherwise)	−0.940 (−1.77)	0.000	0.000	0.004	0.358	−0.361
Subject educational level (1 if a university degree holder; 0 otherwise)	3.197 (3.08)	0.000	0.000	−0.027	−0.840	0.868
Subject occupation (1 if retired; 0 otherwise)	−2.533 (−2.00)	0.000	0.003	0.146	0.493	−0.643
Ophthalmological diagnoses (1 if retinitis pigmentosa; 0 otherwise)	1.579 (2.15)	0.000	0.000	−0.003	−0.542	0.546
Ophthalmological diagnoses (1 if albinism; 0 otherwise)	−2.541 (−2.55)	0.000	0.005	0.189	0.390	−0.584
Standard deviation of the random parameter	2.538 (2.96)					
Ophthalmological diagnoses (1 if other retinal diseases—diabetic retinopathy, ROP, retinal detachment; 0 otherwise)	3.616 (2.89)	0.000	0.000	−0.006	−0.726	0.731
Standard deviation of the random parameter	6.004 (3.27)					
Usage of assistive devices for O and M (1 if filter; 0 otherwise)	1.650 (2.34)	0.000	0.000	-0.003	−0.541	0.544
Standard deviation of the random parameter	2.082 (3.01)					
Visual function characteristics						
Visual acuity (1 if 0.05 ≤ VA < 0.1; 0 otherwise)	2.195 (2.35)	0.000	0.000	−0.005	−0.665	0.670
Contrast sensitivity (1 if poor; 0 otherwise)	2.955 (2.62)	0.000	−0.006	−0.202	−0.510	0.718
Peripheral visual field (1 if horizontal plan ^1^ is restricted and the vertical plan ^2^ is very restricted; 0 otherwise)	8.350 (2.35)	0.000	0.000	−0.004	−0.683	0.686
Mobility characteristics						
Moving about in stores (1 if no difficulty; 0 otherwise)	−3.184 (−2.83)	0.000	0.016	0.307	0.359	−0.682
Walking down steps (1 if extreme difficulty; 0 otherwise)	5.618 (3.12)	0.000	0.000	−0.017	−2.223	2.240
Walking at night (1 if extreme difficulty; 0 otherwise)	2.121 (2.23)	0.000	0.000	−0.013	−0.694	0.707
Fallen incidence (1 if fallen last year; 0 otherwise)	2.301 (2.91)	0.000	−0.001	−0.091	−0.553	0.645
Mobility training (1 if did not undertake any mobility training; 0 otherwise)	2.788 (1.69)	0.000	−0.012	−0.273	−0.286	0.571
Reason for not undertaking mobility training (1 if no environmental adaptation for persons with low vision outside home; 0 otherwise)	−6.980 (−1.97)	0.001	0.975	0.024	−0.438	−0.561
Pursuing out-of-home activities (1 if pursuing two to three out-of-home activities per week; 0 otherwise)	−2.944 (−3.20)	0.000	0.005	0.179	0.557	−0.741
Usage of Apps on smart phone (1 if constantly uses taxi App; 0 otherwise)	2.014 (2.25)	0.000	0.000	−0.004	−0.616	0.619
Challenges when using public transport (1 if non-existence of environmental adaptations for persons with low vision; 0 otherwise)	1.298 (2.09)	0.000	0.000	−0.010	−0.466	0.476
$\mathrm{Threshold}\;\mu_{1}$	5.207 (2.62)					
$\mathrm{Threshold}\;\mu_{2}$	6.900 (3.16)					
$\mathrm{Threshold}\;\mu_{3}$	10.062 (3.50)					
Model statistics						
Log-likelihood at zero	−94.027					
Log-likelihood at convergence	−51.246					
McFadden’s $\rho^{2}$	0.455					
Number of observations	74					
^1^ Horizontal restriction (1) Normal or near normal; 0–20 (2) Restricted; >20–90 (3) Very restricted; >90–160 (4) Extremely restricted; >160–180		^2^ Vertical restriction (1) Normal or near normal; 0–20 (2) Restricted; >20–70 (3) Very restricted; >70–115 (4) Extremely restricted; >115–135				

## Data Availability

Data are unavailable due to privacy.
